# Preferences of Underserved Chilean Women on a Mobile Technology Intervention for Cervical Cancer Screening: Qualitative Study

**DOI:** 10.2196/mhealth.9494

**Published:** 2018-11-20

**Authors:** Mauricio Soto, Javiera Martinez-Gutierrez, McKenzie Momany, Daniel Capurro, Francis Ciampi Spode, Emilia Cea, Tania Mergudich, Klaus Puschel

**Affiliations:** 1 Department of Family Medicine School of Medicine Pontificia Universidad Católica de Chile Santiago Chile; 2 École de Santé Publique Université de Montréal Montreal, QC Canada; 3 School of Medicine University of Washington Seatlle, WA United States; 4 Department of Internal Medicine School of Medicine Pontificia Universidad Católica de Chile Santiago Chile; 5 Centro de Salud Familiar El Roble Santiago Chile; 6 School of Nursing Pontificia Universidad Católica de Chile Santiago Chile; 7 School of Medicine Pontificia Universidad Católica de Chile Santiago Chile

**Keywords:** mHealth, cancer screening, Latina women

## Abstract

**Background:**

In Chile and Latin America, cervical cancer disproportionately affects women of low socioeconomic status. Mobile technology (mobile health, mHealth) may be able to address this disparity by targeting women in underserved populations. However, there is a lack of information regarding barriers to the implementation of mHealth interventions in underserved populations.

**Objective:**

The objective of this study was to investigate the use of cell phones and text messaging (short message service, SMS) in Latina women from disadvantaged communities to design an mHealth intervention for improving cervical cancer screening rates.

**Methods:**

We conducted 9 focus groups among women aged 25-64 years to better understand the implementation barriers and perceptions of a text message (SMS)–based intervention designed to improve cervical cancer screening rates. We used the PRECEDE-PROCEED model to categorize identified themes using template analysis.

**Results:**

Focus group results indicated that older women use mobile phones to receive calls from family and friends but seldom send text messages. Furthermore, they prefer personal contact with their health care providers regarding Papanicolaou (Pap) testing. Younger women, on the other hand, find text messaging easy to use and frequently send texts to family and friends. Importantly, women of all ages mentioned they would like to receive text messages about Pap tests. Factors that facilitate the uptake of the intervention include ease of access to Pap testing, inclusion of family members, and reminder messaging. Potential barriers include cost and the impersonal nature of messaging. Health team members support an mHealth intervention even though they acknowledge the potential barriers to this strategy. Overall, these results support the implementation of an mHealth intervention to increase cervical cancer screening rates.

**Conclusions:**

This study describes the opinions of women nonadherent to Pap testing on the potential use of mobile technologies for cervical cancer screening. Although the overall acceptance was positive, older women prefer personal contact and phone calls over text messaging. Information surrounding these preferences will aid in the implementation of effective strategies to improve cancer screening in underserved populations.

## Introduction

Cervical-uterine cancer is the fourth oncological cause of death in Chile with a mortality rate of 6/100,000 women in 2012. This rate is well above that in developed countries (Canada: 1.71/100,000 women; United States: 2.7/100,000 women) [1]. Studies in Chile have shown marked inequalities in cancer mortality rates between women with low and high educational levels [2]. In Chile, mortality from cervical cancer with respect to education level is 15 times higher in women with <4 years of education than that in women with >12 years of education [3]. Since 1965, the peak cervical cancer screening adherence rate in Chile has been 67%, far from the international recommendation of 80%. Furthermore, Papanicolaou (Pap) testing rates have markedly dropped in recent years, hovering at <60% [4]. There are currently no studies in Chile on inequality in adherence to cervical cancer screening. In Latin America, factors associated with screening adherence include access to health insurance and high socioeconomic status, while factors associated with nonadherence (in women with preinvasive cervical lesions) include concern about the procedure, time constraints, and lack of knowledge. Therefore, it is recommended to develop programs that address these barriers and change communication techniques in a manner that considers broader cultural frameworks [5]. Moreover, recent studies have shown that it is vital to involve community members throughout the program development process in order to maximize effectiveness [6-9].

Interventions based on short message service (SMS) text messaging have shown efficacy in encouraging behavior modification and improving health. Recently, evidence has showed that these interventions have been able to increase the cancer screening adherence [10-12]. A study on Korean American women using a week-long SMS text message intervention demonstrated increased acceptance to cervical cancer screening [13]. However, there is insufficient evidence to characterize maximally effective interventions, and none of these studies have been conducted in Latin American countries. An SMS text message intervention could fail for multiple reasons. Previously described barriers include SMS text message character restrictions, user illiteracy, poor coverage and signal quality, shared cell phones, confidentiality, and message appropriateness [14]. In the United States, the largest users of SMS text messaging are Hispanic people (83%), followed by African-American (76%) and white (70%) people [15], suggesting that cultural factors may influence the use of SMS text messaging. In Chile, there has been a marked growth in the number of mobile phone users in recent years. In December 2015, the number of subscribers reached 23,206,353 people, with a ratio of 128.22 mobile subscribers per 100 inhabitants [16]. According to the same report, an average of 109 million messages were generated per month in 2015, with 99.2% corresponding to SMS text messages and 0.8% to multimedia messaging service messages. These data support the use of SMS text messaging as a strategy to improve cervical cancer screening adherence in Chile. However, no study has yet described mobile phone use by Chilean women. Studying the use of mobile phones would allow us to understand the role they play in women’s lives and enable the design of effective interventions to improve cancer screening adherence.

This study aims to investigate the use of cell phones and SMS text messaging in Latina women from disadvantaged communities in order to design a mobile health (mHealth) intervention to improve cervical cancer screening rates.

## Methods

### Inclusion Criteria

Women aged between 25 and 64 years who were nonadherent to cervical cancer screening guidelines (no Pap test within last 3 years) were included from three health care centers in underserved areas of Santiago. These health care centers are located in the outskirts of the city in the La Pintana and Puente Alto neighborhoods; 30% and 14% of the population, respectively, in these areas lives under the poverty line compared with 9% living under the poverty line in the entire metropolitan area [17].

### Focus Groups

We conducted 9 focus groups in total—3 in each health center; one group of women aged 25-44 years, a second group of those aged 45-64 years, and a third group of health care personnel. Women were divided by age due to potential differences in cell phone use and generational barriers that could affect the use of mHealth interventions. Overall, 27 women participated in the interviews.

In the health professionals focus groups, 10 midwives and 1 paramedic participated. Focus groups were facilitated by 2 researchers trained in qualitative research methods. The interviews were recorded and transcribed verbatim for further analysis.

Topics were investigated through a semistructured guide and included the pattern of cell phones and other mobile use and barriers and facilitators to an SMS text message intervention to encourage Pap smear adherence. Each participant signed an informed consent document to participate in this study. This study was approved by the Ethics Committee of the Universidad Católica de Chile (ID: CEC Med UC 213).

### Data Analysis

The focus group audiorecordings were transcribed and encoded using qualitative data analysis software (Atlas.ti version 6.2). An “analysis by templates” method was used for coding, based on a list of codes derived from the data analysis. This allowed for the creation of broader categories derived from emergent groups of code [18]. Coding was carried out by 2 researchers, using a consensus method to create new categories and resolve differences in the assessments. They identified first-order themes that corresponded to general cell phone and SMS text message use (main uses, barriers, and facilitators to use). In addition, we asked about preferences regarding cervical cancer prevention strategies based on SMS text messaging (usage, content, format, potential barriers, and facilitators). The identified themes were subsequently categorized according to the PRECEED-PROCEED model as factors that could serve as predisposers, facilitators, or reinforcers of SMS text messaging as a means to improve Pap smear adherence [19,20].

## Results

We conducted 6 focus groups with 27 women aged between 25 and 64 years and 3 focus groups with 11 health care workers. Tables 1 and 2 present participants’ demographics.

**Table 1:** Demographics of female focus group participants (N=27).

Characteristic		Value
Age (years), mean (SD)		41 (11.7)
People per household, n (%)		4 (15)
Children per household, n (%)		2 (7)
**Marital status, n (%)**		
	Married	17 (63)
	Single	8 (30)
	Separated or divorced	2 (7)
**Monthly income, n (%)**		
	<US $360	13 (48)
	US $360-800	9 (33)
	US $800-1500	5 (19)
**Education level, n (%)**		
	Completed secondary education	18 (67)
	Incomplete secondary education	6 (22)
	Incomplete primary education	3 (11)
**Occupation, n (%)**		
	Housewife	17 (63)
	Student	3 (11)
	Employed	7 (26)

**Table 2:** Demographics of health professionals (N=11).

Characteristic		Value
Age (years), mean (SD)		34 (9.1)
**Gender, n (%)**		
	Male	4 (36)
	Female	7 (64)
**Occupation, n (%)**		
	Midwife	10 (91)
	Technician	1 (9)
**Education level, n (%)**		
	Postsecondary, nonuniversity	1 (9)
	University	10 (91)

**Identification of the components of the PRECEED-PROCEED model regarding the use of mobile phones for cancer prevention.**

**Predisposing factors**

- Use of technologies for personal and family contact.
- Economic barriers may limit access to mobile technology.
- Mobile phones are most frequently used for calls and messages.
- A significant proportion of older women report low use of phones for making calls and little use of other mobile functions.

**Enabling factors**

- Participants would like to be contacted by the health center, in general, and midwives, in particular.
- Most women have their own mobile phone.
- Theft, loss, or damage to the mobile phone would not be a barrier to maintaining the same cell phone number.

**Reinforcing factors**

- Use of multidimensional messages.
- Use of short and clear messages.

### PRECEDE-PROCEED Model

SMS text messaging knowledge and attitudes, as well as practical knowledge of cell phones and messaging, were considered to be predisposing factors. Facilitating factors were considered to be those that will enable the use of SMS text messaging as a cervical cancer prevention strategy. In addition, the content of messages as well as their frequency and timing were considered to be reinforcing factors for the ongoing use of such SMS text messages as a preventive strategy. Textbox 1 presents an analysis summary.

### Predisposing Factors

#### Cell Phone and Short Message Service Text Message Use

Cell phone usage varied by age. Women in the youngest age group (25-44 years) used their cell phones frequently (multiple times per day), while those in the older age group (45-64 years) reported less usage (the majority used their phones once every 2-3 days).

##### Communication With Family Members

The primary use of cell phones and messaging reported by women of all ages was communication with their families. Within this category, communication with their children was reported as the most frequent use, followed by communication with other relatives such as husband, mother, brothers, or sisters.

I use [my phone] only for communication with my children, and just with text messages. When I am at home with my two children, I don’t use my phone. When my husband gets home, all the more reason [to not use it]. [I use my phone] only [to communicate] with my children.

Participant from Group 1, 25-44-year olds

##### Communication for Work

Communicating for work or school was another important but less frequent use that was brought up only among the younger women. Both groups reported other but less frequent uses, including communication with people outside their family and looking up information.

#### Barriers to Use

Financial considerations were the primary barrier to the implementation of an mHealth intervention. Most participants had prepaid cell phone plans and depended on money to have a functioning mobile phone. One group of women aged 25-44 years comprised women of higher socioeconomic status. These women had plans that included multimedia, allowing them to use additional mobile functions. In the 25 to 44-year-old age group, some women reported having Wi-Fi access at home or in public places. This allowed them to utilize all mobile phone functions more frequently.

It just so happens that in the middle of the month I can’t make calls or send messages if I run out of money.

Participant from Group 6, 45-64-year olds

In the older group, some women indicated that mobile phones were a nuisance and took too much effort to use. In addition, they said that they did not have a thorough understanding of the various functions of cell phones, which was associated with a lower usage.

Look, my children always call me on my cell phone. I sometimes have 8 missed calls. You're not going to believe me, but I don’t answer them. But if you call me on the landline, I answer right away. [I don’t want to give you] false hope that I'm going to read [the messages, because] I won’t.

Participant from Group 2, 45-64-year olds

#### Type of Use

Cell phones were most frequently used for calls, specifically receiving calls. Younger participants used phones for both making and receiving calls, while older participants mainly used them to receive calls. There was a group of women who, despite having a mobile phone and receiving calls, did not answer the calls because they did not notice them or were otherwise occupied. The group of 45 to 64-year-old women expressed being more comfortable with landlines.

Yes, if I have a cell phone, I use it to communicate with my children, but it’s always they who call me. I don’t like using it. I don’t like bringing it everywhere.

Participant from Group 2, 45-64-year olds

Younger participants also sent and received SMS text messages more frequently; older participants used SMS text messaging sparingly. In addition, younger participants appeared to use WhatsApp and other social media, particularly Facebook, for communication. The younger group included women who used WhatsApp rather than SMS text messaging for work and family communication. This same group also used Facebook to keep in contact with distant relatives.

I [use my cell phone] only for communication with my family via WhatsApp or to keep in contact with distant relatives via Facebook. There is very little you can do by making a call on a cell phone.

Participant from Group 1, 25-44-year olds

Those who used SMS text messaging perceived it as a useful form of communication because messages could be personalized and read at one’s convenience.

You can read your messages whenever you want—you’re in control.

Participant from Group 1, 25-44-year olds

### Enabling Factors

#### Contact Preferences

Women from all age groups were favorable toward the idea of being contacted by their health center as part of an mHealth intervention.

I like the idea of messages. I get informational messages at the place I study at, and they’re very good.

Participant from Group 1, 25-44-year olds

Yes, that message [“Go and get your PAP smear” or “did you know that by doing a PAP”...] would be good.

Participant from Group 4, 45-64-year olds

Women across all groups had different preferences and recommendations for the design of an mHealth intervention. Most of the women indicated that they would prefer a call from their health care centers as means of communication. They thought phone calls allowed for more detailed and personalized conversations and had received calls from their health center in the past, for reasons like rescheduling appointments.

I prefer phone calls, because I understand better what they’re trying to tell me.

Participant from Group 5, 25-44-year olds

You can ask questions

Participant from Group 5, 25-44-year olds

Older women rated phone calls over other modes of communication like SMS text messaging and email.

For people like us who aren’t so tech saavy, I think it would be better to use phone calls. For example, her text message got deleted, and others here don’t read them.

Participant from Group 4, 45-64-year olds

I think it would be better if they called because it’s more personal and you would be able to answer and say if you can’t go [to the appointment], need to change the date, etc. With a message, you wouldn’t be able to answer.

Participant from Group 3, 25-44-year olds

On the other hand, younger participants indicated a preference for the use of messages either via SMS text or WhatsApp, and email for more extensive messages.

I think we have more interaction with the midwives now that there is internet and we can contact with them through WhatsApp.

Participant from Group 5, 25-44-year olds

Participants in general did not feel strongly regarding which member of the health care team contacted them as long as he or she was an expert on the subject. However, some participants preferred to be contacted by a midwife, as midwives are well known and perceived to be knowledgeable.

As long as [the call/message] comes from the health center, it’s fine.

Participant from Group 1, 25-44-year olds

[…the message] has to come from the midwife who knows the most. I would think she knows best.

Participant from Group 3, 25-44-year olds

This lack of consensus regarding the preferred contact method (whether via phone call or SMS text message, for example) may reflect different ways in which the interviewees use their mobile phones in daily life. For example, older women who prefer to be contacted via telephone typically communicate with their relatives via telephone calls.

#### Cell Phone Ownership

All women interviewed were the primary users of their cell phone. Some women in both age groups reported sharing their cell phone with children or grandchildren to allow them to play games. Having “dumbphones” (a basic cell phone without smartphone capabilities) allowed women to prevent their children from using them. One woman shared her cell phone with her husband.

Everyone has their own device, so no one needs to share.

Participant from Group 1, 25-44-year olds

Most of the time my children use my phone since it has games on it. They spend the most time with my cell phone.

Participant from Group 3, 25-44-year olds

#### Portability

Most women in the focus groups reported that they had not changed their mobile phone number in years. Some women replaced their cell phones after they were stolen or lost but still managed to keep the same number. This is possible due to a number portability law that allows them to keep their number even if they switch carriers. As such, people tend to believe that they can keep the same number even if their mobile is stolen, lost, or damaged. This practice would facilitate communication between health centers and patients.

I have been robbed twice but kept my same number. They always steal my phone.

Participant from Group 1, 25-44-year olds

It is important to add that recruiting women via phone was one of the study’s biggest difficulties, so the women interviewed here may represent a subgroup that changes its telephone numbers less frequently.

### Enhancing Factors

#### Message Content

Two types of messaging content were identified as useful for women: informational messages and reminder messages.

##### Reminder Messages

Participants were very supportive of the use of messages to remind them if they are due for a Pap test.

I want a reminder because I forget.

Participant from Group 3, 25-44-year olds

It would be very good [to be sent reminder messages]…you would remember that you need to get a PAP and that you have to go tomorrow or the day after tomorrow [to the health care center].

Participant from Group 4, 45-64-year olds

##### Informative Messages

There was a consensus among the groups that it is useful to receive general information on cervical cancer prevention, Pap screening, and common myths.

More than anything, it should have information about PAP tests and emphasize that it doesn’t hurt

Participant from Group 5, 25-44-year olds

[You should] not only call and tell them to get their PAP, but also give information on the consequences if they don’t.

Participant from Group 4, 45-64-year olds

#### Messaging Format

Most women agree that the SMS text messages should be concise and use simple language.

And in simple words, please, in simple words, not technical language

Participant from Group 1, 25-44-year olds

There was less agreement regarding the tone of the messages. One group of women suggested that the messages should use the word *cancer* to have a more serious impact. They mentioned the notion of death should be included to emphasize the consequences of not having regular Pap tests for early detection of cervical cancer. On the other hand, a different group of women thought generating fear could be counterproductive.

PAP smears prevent cancer, and the word “cancer” scares people. It’s good to say “cancer” because we need [to generate] awareness.

Participant from Group 6, 45-64-year olds

### Opinions From Health Professionals Regarding an mHealth Strategy for Cervical Cancer Screening

Pap screening rates are part of Chile’s performance evaluation system in Primary Health Care. Higher rates of screening are associated with more financial resources at the public primary health level. As a consequence, this screening test is highly prioritized in Primary Health Care. Therefore, it is not surprising that interviewees mentioned multiple strategies used by the three centers to improve Pap test adherence. The strategies included having exclusive schedules for Pap testing, daily walk-in appointments, Pap testing in the community, campaigns through posters, and radio announcements, among others. Nonadherent patients are identified when they come to the health center for other services, while in the waiting rooms, and even via telephone calls. In two centers, adherence information from the electronic medical record is used to schedule more efficiently.

Nevertheless, even with these resources allocated for Pap testing, screening rates have not reached the goals set by the Ministry of Health. Health professionals attributed nonadherence to several reasons that can be classified into three major domains: (1) lack of knowledge about the test; (2) negative predisposition toward the test; (3) and misconceptions about the test. Table 3 presents the reasons attributed by health professionals.

When asked about the potential applicability of mobile phones and SMS text messages in cancer screening, professionals of all three centers thought female patients would be receptive to it as the strategy demonstrates concern for their health. However, health professionals were reluctant to believe mobile technologies could help improve Pap test adherence rates unless several barriers were addressed.

In all centers, health professionals believed that women would prefer SMS text messaging as they text frequently and that messages do not need to be read at the same time they are sent. Professionals from one center thought that SMS text messages would be very useful among younger women.

Frequent cell phone number change was the most important barrier identified by professionals in these three centers. From developing scheduling strategies in the past for nonadherent women, the health professionals had determined that they lack a database with updated phone numbers of female patients. Health professionals from two centers mentioned that older women may not know how to use their phones or may have older models that do not properly display messages. In one center, the lack of availability of walk-in appointments for Pap tests was identified as an organizational barrier to addressing Pap nonadherence.

**Table 3:** Reasons for nonadherence to Pap testing according to health professionals (N=11).

Domain and reasons attributed		Health professionals mentioning the domain, n (%)
**Lack of knowledge about Pap tests**		
	“They haven’t had any health problems.”	6 (55)
	“They don’t know when they need to do the test.”	4 (36)
	“They don’t think the test needs to be performed if they aren’t having current sexual relations.”	4 (36)
	“They feel healthy, so they think it is not necessary to carry out the test.”	4 (36)
**Negative predisposition toward the test**		
	“They’re very busy/don’t have time.”	10 (91)
	“They’re always postponing the test.”	5 (45)
**Attitude toward the test**		
	“They think the test is too painful or unpleasant.”	9 (82)
	“They’re too embarrassed by the test.”	6 (55)

Regarding suggestions for an effective strategy using mobile phones and SMS text messages, health professionals recommended the following:

- Use of informative messages with helpful and clarifying information about Pap tests, reminders to get a Pap, available times to get a Pap, and messages that encourage getting a Pap (including information on health risks associated with not getting a Pap)
- Test availability during nonstandard work hours, and the ability to respond to messages to confirm receipt or schedule an appointment

## Discussion

### Principal Findings

This paper discusses the preferences of underserved Latina women on the use of mobile technology for cervical cancer prevention. Although study participants were generally favorable toward the idea of using these technologies, the following points are worth taking into consideration.

Participants primarily use mobile devices to stay in contact with family members. This is similar to what other comparable studies have found. In one study, Hernández et al [21] reported that family care represents one of the most important daily tasks for Mexican women. This finding can be extrapolated to Chilean women and is consistent with the fact that participants primarily use their mobile phones for family contact.

Challenges identified by a World Health Organization study [15] included security and privacy issues, especially in low- and middle-income countries where mobile phones were often shared with family and other community members. In our study, confidentiality did not arise as a barrier to mHealth interventions. Unlike other Latin American women, our participants did not typically share their phones. Regarding mobile usage, phone calls are used more than any other form of communication; this is consistent with how study participants prefer personalized calls from health care providers and also with the literature showing that Latina women use mobile devices for personal communication more than using them for receiving information [22]. In addition, the literature shows that Latina women value the opinions of their family members and health care providers, and these opinions heavily influence their attitudes on cancer screening [23]. This is consistent with the notion of personal contact being important, which was a predominant theme in this study.

Participants in this study preferred short SMS text messages as opposed to longer messages. This aligns with other studies that have identified such SMS text message characteristics as fundamental to the intervention’s success [24,25]. We identified differences in mobile phone use according to participants’ age, as well as variation in the preferred tone of messages. This is consistent with recommendations from the literature to avoid a one-dimensional approach and instead use a variety of messaging types to implement a persuasive health communication strategy [24].

Considering the perspective of both patients and health care teams may aid in the design of a more effective and comprehensive mHealth strategy. This study had many parallels between patients’ and health care personnel’s opinions regarding the potential usefulness, barriers, and recommendations on a mobile strategy to improve Pap screening rates. This may be due to the familiarity that health professionals have developed with women in their health centers.

Regarding health care professionals identifying the frequent change of cell phone numbers as a barrier, although the women in the focus groups did not refer to it as an obstacle, it is important to consider that this was a barrier during the recruitment phase of the study. This should be considered when designing strategies based on mobile phone use.

### Conclusion

Low cervical cancer screening rates remain a public health problem worldwide. Mobile technologies have the potential to improve cancer screening rates in low-income countries, but there is a lack of information on the usefulness of mHealth for cervical cancer prevention in Latin America. Latina women use mobile devices regularly to communicate with their families and value personal contact or phone calls from health personnel; therefore, any successful intervention using mHealth needs to take these factors into consideration. This study will provide valuable information in the design and implementation of a mobile intervention to increase cervical cancer screening rates.

## Abbreviations

mHealth: mobile health
PAP: Papanicolaou
SMS: short message service
